# Affective emotion increases heart rate variability and activates left dorsolateral prefrontal cortex in post-traumatic growth

**DOI:** 10.1038/s41598-017-16890-5

**Published:** 2017-11-30

**Authors:** Chuguang Wei, Jin Han, Yuqing Zhang, Walter Hannak, Yanyan Dai, Zhengkui Liu

**Affiliations:** 1 Key Laboratory of Mental Health, Institute of Psychology, Chinese Academy of Sciences, Beijing, 100101 China; 2 Department of psychology, University of Chinese Academy of Sciences, Beijing, 100049 China; 3 Core Facilities of Institute of Psychology, Chinese Academy of Sciences, Beijing, 100101 China; 4 China Meteorological Administration Training Centre, Beijing, 100083 China; 5 Beijing Polytechnic, Beijing, 100176 China

## Abstract

The present study evaluated the activities of heart rate variability (HRV) and dorsolateral prefrontal cortex (DLPFC) in response to the presentation of affective pictures correlated with posttraumatic growth (PTG) among adults exposed to the Tianjin explosion incident. The participants who were directly involved in the Tianjin explosions were divided into control, post-traumatic stress disorder (PTSD) and PTG group according to the scores of PTSD Checklist-Civilian Version and PTG Inventory survey. All participants received exposure to affective images. Electrocardiogram recording took place during the process for the purpose of analyzing HRV. Meanwhile, functional near-infrared spectroscopy (fNIRS) was used to measure DLPFC activity through hemodynamic response. Our results indicated that, while performing the negative and positive picture stimulating, PTG increased both in low and high frequency components of HRV compared with the control group, but PTSD was not observed in this phenomenon. Moreover, the fNIRS data revealed that PTG had an increased activation in the left DLPFC compared to the control in the condition of negative pictures stimulating, wheras PTSD showed a higher activation in the right DLPFC while receiving positive pictures stimulating. To our knowledge, this is the first study which provides the differences between PTSD and PTG in emotional regulation.

## Introduction

Life-threatening illnesses and events such as earthquakes, motor vehicle accidents or terror incidents may cause post-traumatic stress disorders (PTSD) and post-traumatic growth (PTG)^1–5^. PTG refers to the experience of positive change that occurs as a result of the struggle with highly challenging life crises, describing the experience of individuals who do not only recover from trauma, but also discover it as an opportunity for further individual development. Those individuals overcome trauma with improved psychological functioning in specific domains^6^. PTG has been reported by a significant number of people who have encountered major life challenges, resulting in such factors described as new possibilities, relating to others, personal strength, spiritual change, and appreciation of life^7^.

Traumatic events can cause a series of strong emotional responses, such as fear, anxiety and depression. Since the development of a new chapter in DSM-V on Trauma-and Stress–Related Disorders much more attention has been paid to the emotional symptoms that accompany PTSD^8^. Emotion regulation is defined as a process by which individuals modify their emotional experiences, expressions, and subsequent physiological responses^9^. Meanwhile, emotion regulation is recognized as a facet of coping which is central to trauma recovery and represents an important ability to manage, experience, and express intense negative feelings associated with traumatic events and thereby coping with trauma^10^. In this sense, emotion regulation to traumatic reminders equals a hallmark phenomenon during the post-traumatic experience, and the changes of emotion regulation which including the emotional experiences and expressions are central features of the long-term psychological consequences of traumatic events.

Many studies across both clinical and non-clinical research have provided evidence that emotion dysregulation is associated with PTSD across ethnically diverse samples. Nicole H. Weiss *et al*. declared that emotion dysregulation may contribute to the development, maintenance, and/or exacerbation of PTSD among substance use disorder patients with PTSD^11^. Natalie R. Stevens *et al*. found that child abuse exerted a direct effect on post-traumatic symptoms and indirect effects through difficulties with emotion regulation^12^. Ehring and Quack used questionnaires to assess characteristics of emotion regulation, showing that PTSD symptom severity was significantly associated with all variables assessing emotion regulation difficulties^13^. Furthermore, Matthew T. Tull *et al*. declared that PTSD symptom severity was found to be associated with lack of emotional acceptance, limited access to effective emotion regulation strategies, and lack of emotional clarity. Meanwhile, overall difficulties in emotion regulation were associated with PTSD symptom severity, controlling for negative affect^14^.

However, remarkably few studies examined the emotion regulation associated with PTG. Larsen and Berenbaum found that emotion expressive suppression positively predicted distress, but not PTG. However, bootstrapped mediation models showed that emotional processing has a significant indirect effect on PTG and distress through its effect on creating meaning^15^. Researchers examine the mediating effects of perceived social support, hopefulness, and emotional regulation on the relationship between enacted stigma and PTG among HIV-affected children, and the results showed that emotional regulation together with hopefulness and perceived social support mediated the impact of enacted stigma on PTG^16^.

## Heart rate variability

The emotion regulation that humans experience while interacting with the trauma is associated with a high degree of physiological arousal. Indeed, acute and chronic psychosocial stress not only increases heart rate but also significantly reduces heart rate variability (HRV) in humans^17^. The power spectrum of short-term time series contains two major components of high frequency (HF, 0.15–0.40 Hz) and low frequency (LF, 0.01–0.15 Hz). HF-HRV and LF-HRV reflecting parasympathetic (vagal) activity and baroreflex function respectively^18,19^. HRV is common across anxiety-related phenomena and have a relation with vagal tone, state, trait, and clinical forms of anxiety, and represents a reliable and important psychophysiological marker of emotion regulation capacity. Individuals with greater emotion regulation ability have been shown to have higher levels of HRV^20^. Moreover, the LH-HRV and HF-HRV components have different contribution during the emotion regulation processes. Comparing with LF-HRV component, the HF-HRV component is considered to play a much more important role in these processes. Specifically, HF-HRV will increase in the emotion regulation condition relative to simply an emotion-induced condition, which means a higher cognitive control ability and more activity of the parasympathetic nervous system in decreasing the emotion response^21^. Meanwhile, it has been proposed that LF-HRV play an important role in anxiety-related emotional control, high LF-HRV has been linked to higher anxiety level, and less emotional control ability^22^.

Some evidence in the literature revealed that LF-HRV and HF-HRV were most often reported lower in people with PTSD compared with healthy controls and trauma-exposed individuals without PTSD. The research by Wahbeh showed that LF-HRV and HF-HRV were lower in combat veterans than comparing no-PTSD with PTSD^23^. Moreover, Hauschildt *et al*. found that PTSD showed lower LF-HRV and HF-HRV than non-trauma-exposed controls at baseline and throughout different affective conditions^24^.

To our knowledge, however, there is no research on the relation of HRV and PTG.

## Dorsolateral prefrontal cortex

Executive brain areas, especially the prefrontal cortex (PFC), exert an inhibitory influence on sub-cortical structures, such as the amygdala, allowing the organism to adaptively respond to demands from the environment, and organize their emotional and behavioral responses effectively. Thus, active cortical brain areas are indicative of greater inhibitory and emotion regulation^20^.

There is a growing body of literature which suggests that individual differences in HRV are related to PFC activity. In addition, it should be noted that the dorsolateral prefrontal cortex (DLPFC) may play a more important role because of its unique anatomy. HRV was associated with activity patterns in the ventromedial prefrontal cortex (vmPFC), individuals with higher HRV showed both higher overall vmPFC blood-oxygen-level-dependent activity^25^. Sakreida *et al*. reported the effects in HRV by manipulating the cortical excitability of the DLPFC through neuro-navigated repetitive low frequency transcranial magnetic stimulation (rTMS)^26^. DLPFC showed a higher positive co-variation between HF-HRV and regional cerebral blood flow^27^. Moreover, some research confirmed that the left and right DLPFC contribute differently to the LF and HF of HRV. Lane *et al*. declared that HF correlated with blood flow in the right DLPFC^28^, while LF is also affected more by DLPFC. Gonçalves applied tDCS over the left DLPFC and found a decrease in LF^29^.

DLPFC also has been proposed to be a central regulatory brain area in emotion processing. Although DLPFC has no direct anatomical connection to the amygdala, it regulates an emotion generated by amygdala, insula and ventrolateral prefrontal cortex, therefore it might exert a more indirect control over areas of affect generation by its projections to the pre-supplementary motor area and anterior midcingulate cortex, which are both involved in emotion regulation and have an association to regulation success^30^.

Because of the important roles of DLPFC in HRV and emotion function, it becomes a region of interest which involved the pathophysiology of PTSD. Aupperle *et al*. used functional magnetic resonance imaging (fMRI) to examine neural responses during anticipation of negative and positive emotional images in PTSD. They found that greater DLPFC activation was associated with lower PTSD symptom severity^31^. Similarly, a longitudinal neuroimaging study reported that greater DLPFC thickness was associated with greater post-traumatic stress disorder symptom reductions and better recovery^32^. Moreover, a clinical research study reported that PTSD patients who received 10 daily sessions of right dorsolateral prefrontal rTMS at a frequency of 10 Hz experienced greater therapeutic effects than with slow-frequency application or sham stimulation^33^. This implied that the DLPFC region is not only involved in PTSD, but might also correlate closely with psychological recovery, such as PTG, from a severely traumatic event in humans.

While there is a great deal of research focusing on neural correlations of PTSD, the research on PTG has received much less attention compared with PTSD, and the work on neural correlation with PTG is very scarce. As a result, PTG is an appealing but poorly understood construct. We used Google scholar and Pubmed to search for research on neural correlation of PTG, and found only two publications. Research by Rabe demonstrated that greater relative left baseline prefrontal activation measured by EEG alpha power corresponded with greater self-perceived PTG, as reflected in the post-traumatic growth inventory (PTGI) scale. Moreover, relative left fronto-central activity was associated with the PTGI dimensions of new possibilities, changed relationships, appreciation of life, and personal strength but not with spiritual changes^34^. A very recent fMRI study showed that the total scores on a PTGI were positively and significantly associated with the delta-regional grey matter volume in the right DLPFC, indicating that the DLPFC seems to be the main neural correlate of PTG^35^.

Therefore, we intended to explore the changes of HRV and the activation of the DLPFC accompanied emotion experience in PTG. We compared the characteristics of these correlated physiological and neural changes between PTG and PTSD. Our primary interests included: (1) Comparison of differences in HRV between PTG and PTSD while performing affective pictures; (2) Exploration whether hemoglobin concentration changes in the DLPFC modulated by affective pictures are different between PTG and PTSD. We hypothesize that the low and high frequency components of HRV in PTG may be higher while performing affective pictures, and there may be a differentiation of emotion function in DLPFC between PTG and PTSD.

## Materials and Method

### Participants

On Wednesday, 12 August 2015, a series of explosions that killed 165 people and injured hundreds of others occurred at a container storage station at the Port of Tianjin. Initially, for the purpose of investigating the HRV changes while performing affective picture stimulating among PTSD and PTG subjects, a sample of 90 participants was collected from central and surrounding areas affected very seriously by the explosions in Tianjin. Then, in order to explore the activation of the DLPFC, all participants were asked to receive fNIRS measurement through the same selection route. The basic information of the samples is presented in Table 1. All participants experienced the explosions within 3 kilometers.

**Table 1:** Basic information of participants.

	Sample (n = 90)	
	N	%
**Gender**		
Male	53	58.89
Female	37	41.11
Age (M ± SD)	28.58 ± 6.52	
**Educational level**		
Less than high school	8	8.89
High school	36	40.00
More than high school	46	51.11
**Marital status**		
Married	51	56.67
Other	39	43.33

### Ethics statement

This study was approved by the ethics committee of the Institute of Psychology, Chinese Academy of Sciences. All methods and protocols in the experiment were performed in accordance with the relevant guidelines and regulations of the approved methods and protocols. The procedure of the study was fully explained to the participants, and informed written consent was obtained from each participant before the study.

### Psychological Assessment

The participants were recruited and asked to complete a set of questionnaires, including the PTSD Checklist-Civilian Version (PCL-C), Posttraumatic Growth Inventory (PTGI). Details of the measures will be further introduced as follows.

PCL-C is an easily administered self-report measure, and consists of 17 items which correspond directly to DSM-IV PTSD symptoms. Each item is rated on a five-point Likert scale using anchors ranging from one “not at all” to five “extremely,” reflecting the extent to which the particular symptom disturbed the respondent during the past month^36^. The Chinese version of the PCL was adapted by a stringent two-stage process of translation and back translation. Adequate levels of internal consistency (Cronbach’s α above. 77) have been previously reported for the total scale and three subscales^37^. In this study Cronbach’s α for the scale was 0.928 and participants of the sample were instructed to complete the PCL-C referring to the “explosions of Tianjin.”

PTGI was developed by Tedeschi and Calhoun to assess perceived positive changes after trauma. The inventory consists of five subscales comprising 21 items: personal strength, new possibilities, relating to others, appreciation of life, and spiritual change^7^. The Chinese version of PTGI was developed through translation and back-translation. This version is a six-point scale ranging from 0 (I did not experience this change after the traumatic event) to 5 (I experienced this change to a very high degree after the traumatic event). The internal consistency of mean PTGI scores was very good in the sample (21 items, α = 0.939).

PCL-C score of 38 points was considered as the cutoff value for PTSD diagnosis in the actual screening^38^. In the analysis of PTGI scores, a histogram was used to examine logical cut-off scores for participants identified as “high” and “low” growth reporters. The mean for the sample was 63.62, and “high” growth reporters were identified as those with scores of 80 or higher. All participants were divided into three groups according to their scores of PCL-C and PTGI: 1) In the control group (n = 30), scores of PCL-C and PTGI were both lower than the cut-off scores. Mean_PCL-C_ = 27.10, SD_PCL-C_ = 4.91, Mean_PTGI_ = 49.50, SD_PTGI_ = 16.88; 2) In the PTSD group (n = 30), the participants scored higher than 38 in PCL-C but lower than 80 in PTGI, Mean_PCL-C_ = 43.31, SD_PCL-C_ = 7.61, Mean_PTGI_ = 53.01, SD_PTGI_ = 9.04; 3) In the PTG group (n = 30), compared with the PTSD group, the PCL-C’s scores were lower than 38 but the PTGI’s scores were higher than 80, Mean_PCL-C_ = 25.03, SD_PCL-C_ = 5.84, Mean_PTGI_ = 88.35, SD_PTGI_ = 7.69. The details of the groups are presented in Table 2. The scores of PCL-C and PTGI among groups are presented in Table 2.

**Table 2:** The mean scores and SD of PCL-C and PTGI among control, PTSD and PTG group.

Group	Survey	Mean	SD
CON (n = 30)	PCL-C	27.10	4.91
	PTGI	49.50	16.88
PTSD (n = 30)	PCL-C	43.31	7.61
	PTGI	53.01	9.04
PTG (n = 30)	PCL-C	25.03	5.84
	PTGI	88.35	7.69

### Materials and Procedures

45 affective pictures that included 15 pleasant pictures (valence < 3; arousal > 4), 15 unpleasant pictures (valence < 3; arousal > 6) and 15 neutral pictures (valence 4.5–5.5; arousal < 3) were selected from the International Affective Picture System (IAPS, Centre for the Psychophysiological Study of Emotion and Attention, 1994). All pictures were adjusted by Photoshop to keep the same size (768 × 576 pixels) in height and width. Participants were sitting on a chair in a quiet room, The affective pictures were presented via E-Prime software (E-Prime 2.0, Psychology Software Tools, Inc., Pittsburgh, PA, USA) on a 21.5 in. display monitor, 70 cm away from each subject’s face (viewing angle 24°). A white cross hair was first presented at the center of a black background for 20 s (resting period) and then a stimulation that consisted of 3 affective pictures was shown for 45 s (stimulation period, included 1 pleasant, 1 unpleasant and 1 neutral picture, each picture was shown for 15 s). The stimulation period was successively followed by the second resting period. There were 5 resting periods and 5 stimulation periods in total. The order of presenting pictures was randomized in each stimulation period.

The participants were asked to sit during ECG and fNIRS data acquisition. The physiological data were acquired using Biopac MP150 for Windows (Biopac Systems, Inc., Aero Camino, CA) and stored on the hard drive of a Windows XP laptop. Heart rate data were acquired using the electrocardiogram (ECG) modules of the Biopac system. The ECG signal was amplified by a gain of 1000, filtered with a Hamming windowing function, and with a 60-Hz notch filter. ECG was recorded using two disposable Ag/AgCl electrodes pre-coated with electrolyte gel: one was placed on the right side of the upper torso, 1 cm below the clavicle; and the second, on the inside of the left wrist. All data were sampled at 1,000 Hz, digitized at 16 bit A/D resolution, and amplified using the respective modules of the Biopac system.

**Figure 1:**
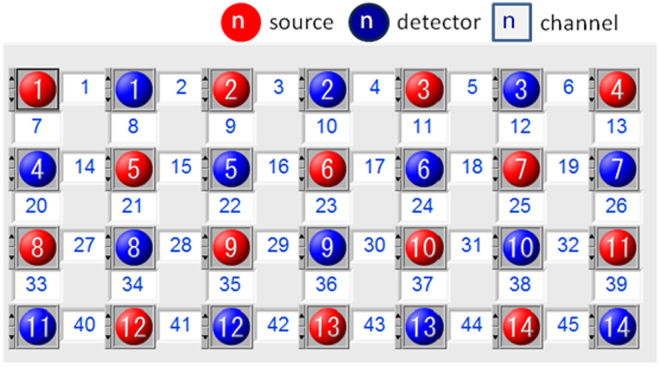
Labels of fNIRS probes and channels.

**Figure 2:**
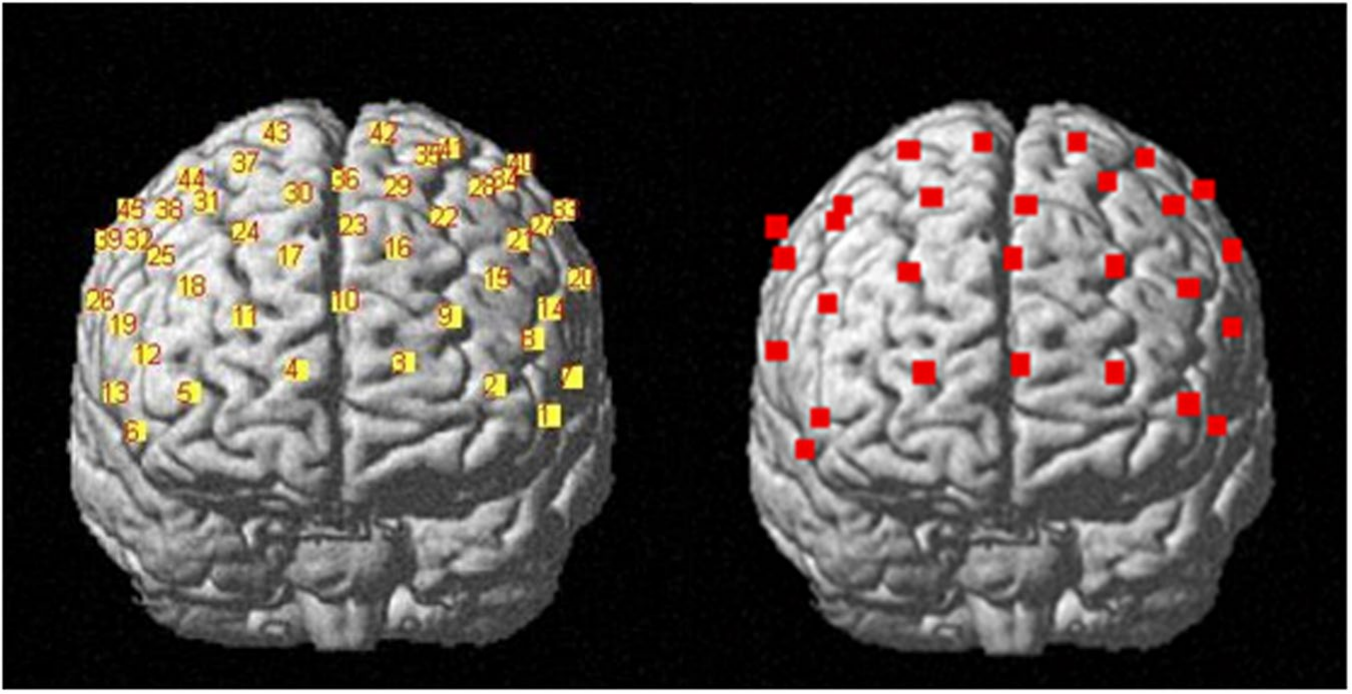
Channel positions rendered onto the cortical surface of the MNI standard brain.

In order to collect fNIRS data, a 52-channel NIRS instrument (LABNIRS, Shimadzu) was used to measure relative concentrations of [oxy-Hb] and [deoxy-Hb]. The instrument used three wavelengths of the near-infrared light (780, 805 and 830 nm). They were combined with a beam coupler and led to the surface of the scalp by a flexible 1 mm diameter optical fiber (NIRS probe). The distance between the source and detector probes was set at 30 mm, and within that distance the instrument could measure the area at 20–30 mm below the scalp, which corresponds to the surface of the cerebral cortices^39^. A plastic hat with probes was carefully fixed on the head of the participants, symmetrically using an elastic band. The size of the detective area was 12 cm × 20 cm, and on the hat 28 probes (14 sources and 14 detectors) were arranged in a matrix pattern, 30 mm apart from each other. The most anterior row of probes was on the Fp1–Fp2 line following the international 10/20 system used in electroencephalography. The probes on the shell measured the relative concentration of oxygenated hemoglobin at 45 points on the subject’s forehead areas. In total, 21 left anterior and 21 right anterior channels were averaged to measure the activities in the left and right PFC respectively (Fig. 1). The time resolution was set at 0.1 s. To seek the cortical loci responsible for the observed hemoglobin concentration changes, NIRS probe and channel locations were registered to the Montreal Neurological Institute (MNI) space. First, five anatomical landmarks (nasion, inion, Cz, left and right preauricular points) and all NIRS probes were digitized using a three-dimensional digitizer (FASTRAK, Polhemus, Vermont, USA). Coordinates of channels in the real space were calculated simply as intermediate points between sources and detectors. Then, the NIRS probe and channel positions were rendered onto the MNI standard brain using the NFRI toolbox^40^ implemented in the NIRS-SPM software^41^. Coordinates obtained from individuals in the MNI space were averaged across the subjects (Fig. 2). Finally, each participant’s coordinates were exported and saved as Brodmann’s areas (BA) txt.

### Data Analysis

#### HRV data analysis

First, all data were visually inspected prior to analysis, any ectopic/non-sinus beats, such as atrial tachycardia, ventricular tachycardia, and atrial premature beats, were identified and removed after visual inspection. Meanwhile, the motion artifacts were isolated through independent component analysis. Second, the power of the low and high frequency components were evaluated by using the analysis function for heart rate variability of Acknowledge 4.2 software. Third, measures of the low and high frequency of HRV data were natural-log transformed to normalize their skewed distributions. Finally, all statistical data were coded and entered into SPSS for windows version 20 (IBM SPSS Statistics), One-way analysis of variance (ANOVA) was employed to analyze emotion effects in three emotion conditions (negative, neutral and positive) among three groups.

#### Oxygenated hemoglobin analysis

Relative changes in oxygenated hemoglobin were analyzed using procedures previously developed by Plichta *et al*.^42^ programmed in Matlab (2013a, The MathWorks Inc., MA,USA) which by and large are comparable to typical fMRI analysis based on the general linear model (GLM) framework. In order to improve the signal-to-noise ratio, a highpass filter based on a discrete cosine transform was employed. Beta values of event-triggered signals were obtained through the GLM, and then the betas of the DLPFC channel (overlap DLPFC area more than 50%) were averaged among the subjects within three groups (control, PTSD and PTG) and under three emotion conditions (negative, neutral and positive). At last, mean values of these DLPFC betas were analyzed by means of repeated measures ANOVA. Data are provided as means of beta × 10^3^ (unitless) throughout the text. Statistical analyses were conducted with SPSS for windows version 20 (IBM SPSS Statistics).

## Results

**Figure 3:**
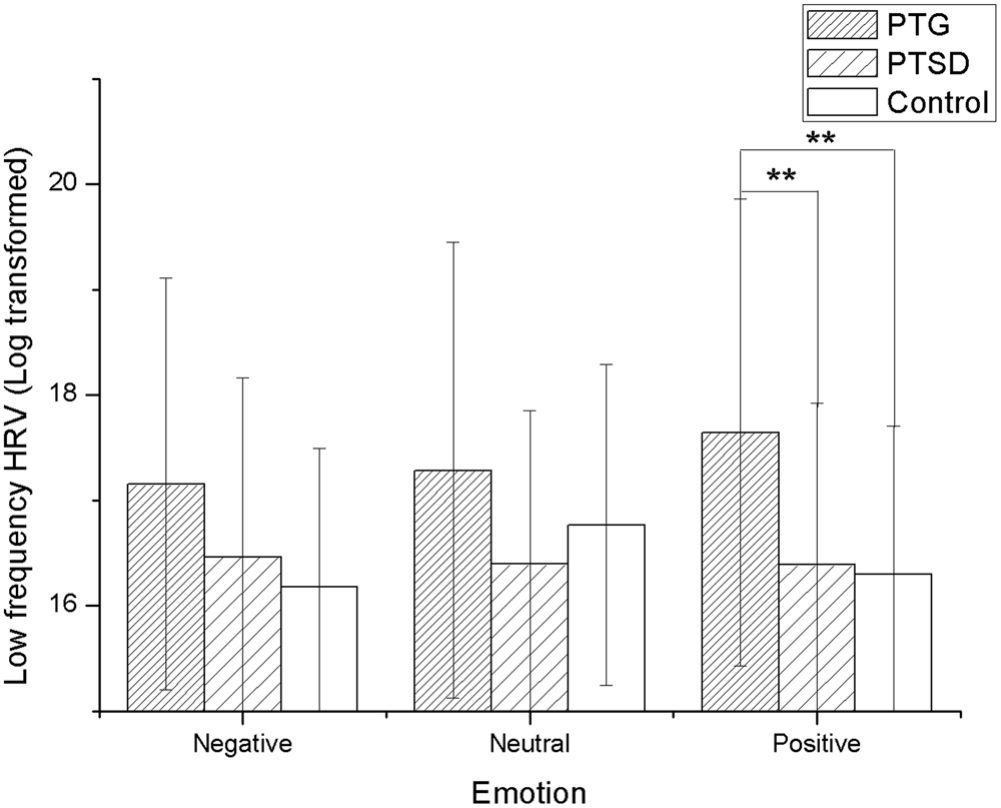
Significant group differences in the negative and positive affective pictures stimulating between the control group and the PTG group in the low frequency HRV, which showed that the PTG group had higher low frequency HRV *p < 0.05, **p < 0.01.

One-way ANOVA revealed a significant difference among groups for the power of low frequency component in negative [F(2, 89) = 2.675; p = 0.045] and positive [F(2, 89) = 5.499; p = 0.006] affective stimulating conditions, but no difference in neutral [F(2, 89) = 1.965; p = 0.146] condition. Bonferroni multiple test corrections for the ANOVA post-hoc comparisons suggested that while performing negative pictures, there was no difference for the power of low frequency between PTG and control group (p = 0.027 > 0.025). Moreover, no difference was found between PTG and PTSD group (p = 0.114 > 0.025), no difference was found between PTSD and control groups (p = 0.515 > 0.025). Meanwhile, Bonferroni multiple test corrections revealed that while performing positive pictures, the power of low frequency of PTG was higher than control group (p = 0.004 < 0.025) and PTSD group (p = 0.007 < 0.025), but there was no difference between PTSD and control group (p = 0.844 > 0.025) (Fig. 3).

**Figure 4:**
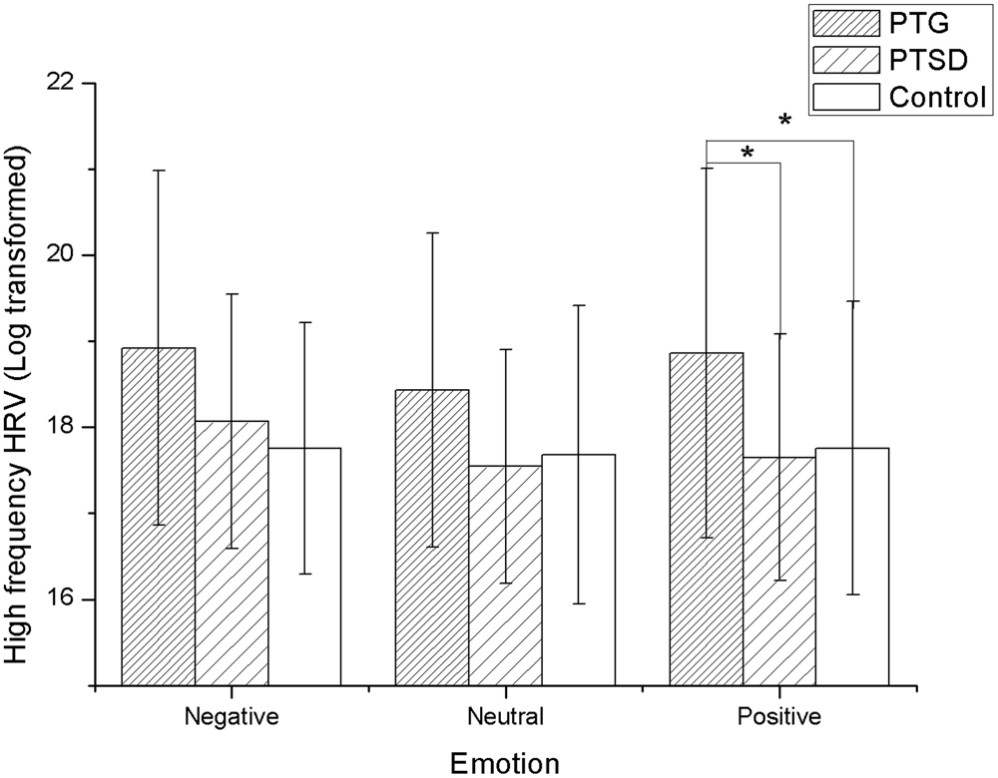
Significant group differences in the negative and positive affective pictures stimulating between the control group and the PTG group in the high frequency HRV, which showed that the PTG group had higher high frequency HRV *p < 0.05.

A significant difference was also found among the groups for the power of high frequency component while performing negative [F(2, 89) = 2.519; p = 0.046] and positive[F(2, 89) = 4.222; p = 0.018] pictures by the one-way ANOVA analysis, but no difference was found in neutral [F(2, 89) = 1.557; p = 0.217] condition. Bonferroni multiple test corrections showed that there was no difference for the power of high frequency between PTG and control group (p = 0.040 > 0.025) in the condition of negative pictures stimulating, and no difference was found between PTG and PTSD group (p = 0.080 > 0.025). Meanwhile, PTSD had no difference compare with control group (p = 0.756 > 0.025). However, while performing positive pictures, the power of high frequency of PTG was higher than control group (p = 0.019 < 0.025) and PTSD group (p = 0.010 < 0.025), but no difference was observed between PTSD and control groups (p = 0.815 > 0.025). The result of high frequency is shown in Fig. 4.

**Figure 5:**
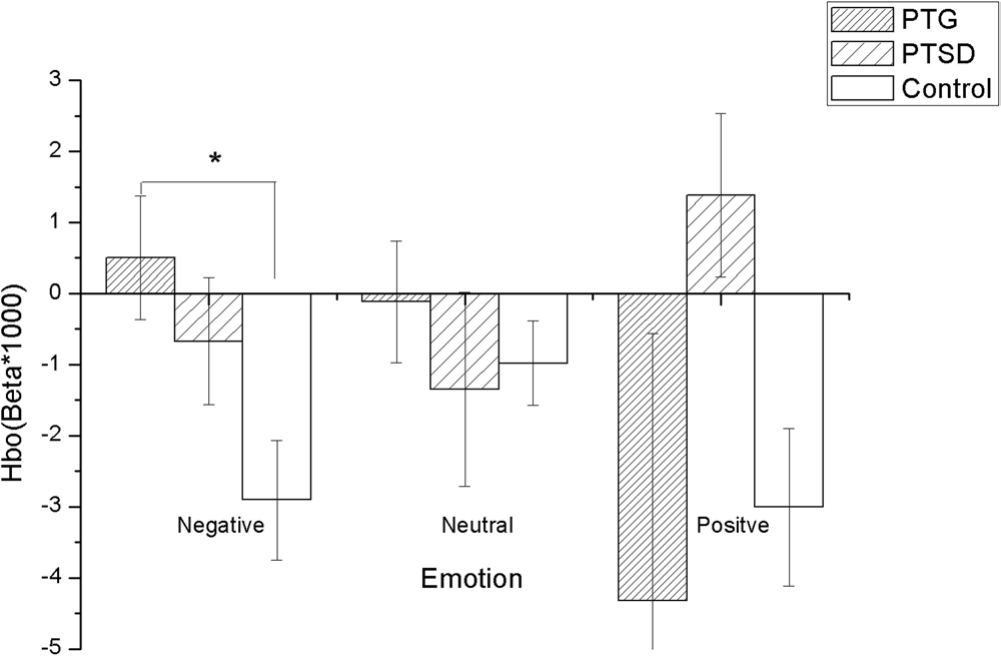
PTG showed a higher HbO (oxygenated hemoglobin) level of the left DLPFC in the negative affective pictures stimulating than the control group *p < 0.05.

**Figure 6:**
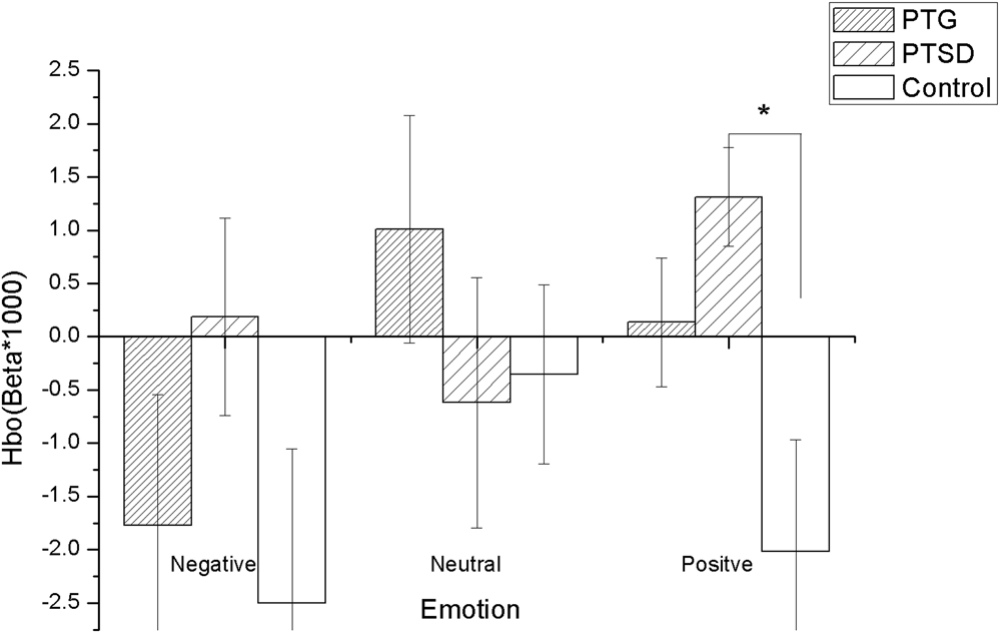
PTSD showed a higher HbO (oxygenated hemoglobin) level of the right DLPFC in the positive affective pictures stimulating than the control group *p < 0.05.

The negative, neutral and positive betas of left and right DLPFC among three groups were analyzed by the repeated measures ANOVA analysis. The results showed that there is a significant overall difference between groups in left (F(2,87) = 4.960, p = 0.009) and right DLPFC (F(2,87) = 7.859, p = 0.001) while performing affective pictures, but no significant effect of (emotion × group) interaction in left (F(2,87) = 1.889, p = 0.157) and right DLPFC (F(2,87) = 1.276, p = 0.284). We applied Bonferroni multiple test corrections for the ANOVA post-hoc comparisons, the results revealed that the oxygenated hemoglobin level of left DLPFC in PTG is higher than in the control group while performing negative affective pictures (P = 0.004 < 0.025), but no difference was found in PTSD (p = 0.070 > 0.025) (Fig. 5). In contrast, the Bonferroni multiple test corrections for the ANOVA post-hoc comparisons showed an increased oxygenated hemoglobin level of the right DLPFC in PTSD while performing positive affective pictures (P = 0.013 < 0.025), but no difference was found in PTG (P = 0.256 > 0.025) (Fig. 6).

## Discussion

The ability of emotion regulation is related to a number of important psychological outcomes, such as attention, decision making, memory, physiological responses, and social interactions^43^. Moreover, emotion regulation has an important role in posttraumatic experience, and the processes of both PTSD and PTG might be influenced by emotional experiences, responses and expressions. HRV is a sensitive and important psychophysiological index of emotion regulation capacity, so HRV has been employed often in PTSD research as an important marker of emotion regulation in trauma. Moreover, HF-HRV is mediated by parasympathetic (vagal) activity while LF-HRV is regulated by baroreflex function^18^.

Our study firstly investigated the characteristics of HRV among PTG and PTSD in response to the presentation of affective pictures. The results demonstrated that the low and high frequency components of HRV in PTG were significantly higher than control group while performing positive affective pictures stimulating. However, we did not observe any significant difference between PTG and the control groups during negative and neutral affective pictures stimulating. These results of HRV experiment suggested that PTG having higher vagal function may allow persons to more efficiently process emotional stimuli.

Although some published literatures have clearly shown that PTSD often had a lower HF-HRV than control, our results indicated that PTSD has no differences of HF-HRV when compared with the control group in response to different affective conditions. The reason could be that the population of PTSD participants is heterogeneous and complicated, with different traumatic events (e.g. war, disaster, terrorist assault) and demographics (e.g. sex, age, religion)^44^. We believe that these factors may have impacted on the relationship between PTSD and HRV.

Activities in prefrontal cortex correlates with vagal function of heart rate variability during emotion regulation. Based on this, our research explored the role of the prefrontal cortex in emotional experiencing between PTG and PTSD. Discovering the different characteristics of emotional experiencing in PTG and PTSD, the results demonstrated that the left DLPFC is more involved in the negative affective experiencing in PTG, whereas the right DLPFC is more involved in the positive affective experiencing in PTSD. The DLPFC is widely concerned because of its overrepresentation in cognitive process such as reappraisal, expressive suppression, working memory, reasoning, cognition and emotion processing, which regulate many behavioral reactions, such as motor behaviors^45^, approach and avoidance via its connections to the other brain areas, such as the ventral striatum, amygdala and insular *et al*.^46^. Many studies revealed that DLPFC is closely involved post-traumatic experience. On one hand, DLPFC plays an important role in the recovery from PTSD, and greater DLPFC response in PTSD patients may reflect engagement of cognitive control networks that are beneficial for emotional and cognitive functioning^32^. On the other hand, the delta- regional grey matter volume in DLPFC is positively and significantly associated with PTG, and DLPFC seems to be the main neural correlate of PTG^35^. These results showed that DLPFC area is not only involved in PTSD, but might also correlate closely with PTG. The present study implied that DLPFC has lateralization effects in the involvement of PTSD and PTG, and future study is needed to further answer the roles of bilateral parts of the DLPFC in maintaining emotional and cognitive aspects of PTSD and PTG.

Based on the above statement, our findings in total suggested an important relationship between the emotion regulation, HRV, and prefrontal cortex function, provided the evidence that prefrontal cortex has a regulatory role in autonomic nerve system during emotion regulation correlate with posttraumatic experience. A considerable amount of studies on PTSD covered many fields during the last decades. As a result, rapid progress in our understanding of psychopathological conditions of PTSD has been made due to the vast research of neural and other biological correlates of these phenomena. PTG represents the experience of positive change which occurs as a result of the struggle with highly challenging life crises. It is manifested in a variety of ways, including an increased appreciation for life in general, more meaningful interpersonal relationships, an increased sense of personal strength, changed priorities, and a richer existential and spiritual life^6^. During the past decades the research on PTG mostly focused on the correlation with trauma (such as traumatic exposure), personality trait (such as resilience, rumination and cognitive coping)^47,48^ and social support^49^. However, to date there is extremely little research on the neuro-biological mechanisms of PTG compared with PTSD. As a result, PTG remains poorly understood and controversial. Because current studies on PTG are mostly dependent on self report some researchers even doubt that the fact that some people seem to grow after trauma, and may deem this illusory^50^. The present study, to the best of our knowledge, is the first study to investigate PTG from the perspective of HRV and hemodynamic responses, and provides more objective evidence to support that PTG is an objective phenomenon.

Of course, there are several limitations in this study. Firstly, the method of group division maybe inappropriate. In this study, we divided the participants into control, PTSD and PTG groups according to their scores of PCL-C and PTGI surveys, taking the view that PTSD and PTG are two opposite outcomes of trauma. However, the correlation between PTSD and PTG still remains controversial. Although our previous research found no correlation between PTSD and PTG^51^, some studies hold the point of view that there is a negative correlation between them and they may coexist^48,52^, while other research suggested that PTSD is positively correlated with PTG^53,54^. In this regard, there might be some individuals who would score highly both in PCL-C and PTGI, and this group was not investigated in this study because of limited participants. Secondly, this research is a cross-sectional study, and the data collection was conducted approximately 12 months after the serious explosions. Therefore, to a certain degree, the characteristics of the emotional experiencing we have drawn from the data is isolated and incomplete, and a retrospective and longitudinal investigation is needed to overcome this shortage. Thirdly, the sample size we recruited was relatively small, which might influence the generalization of the conclusion. Moreover, fitness might be a potential confounding factor influencing the performance of HRV. In future studies, the assessment of fitness should be considered and the relevant index reflecting aerobic fitness, such as total physical activity and maximal oxygen consumption (VO2max), should be collected.
